# Magnetic Superporous Poly(2-hydroxyethyl methacrylate) Hydrogel Scaffolds for Bone Tissue Engineering

**DOI:** 10.3390/polym13111871

**Published:** 2021-06-04

**Authors:** Beata A. Zasońska, Antonín Brož, Miroslav Šlouf, Jiří Hodan, Eduard Petrovský, Helena Hlídková, Daniel Horák

**Affiliations:** 1 Institute of Macromolecular Chemistry CAS, Heyrovského nám. 2, 162 06 Prague 6, Czech Republic; zasonska@imc.cas.cz (B.A.Z.); slouf@imc.cas.cz (M.Š.); hodan@imc.cas.cz (J.H.); hlidkova@imc.cas.cz (H.H.); 2 Institute of Physiology CAS, Vídeňská 1083, 142 20 Prague 4, Czech Republic; Antonin.Broz@fgu.cas.cz; 3 Geophysical Institute CAS, Boční II 1401, 141 31 Prague 4, Czech Republic; edp@ig.cas.cz

**Keywords:** poly(2-hydroxyethyl methacrylate), superporous, scaffold, magnetic, SAOS-2 cells

## Abstract

Magnetic maghemite (γ-Fe_2_O_3_) nanoparticles obtained by a coprecipitation of iron chlorides were dispersed in superporous poly(2-hydroxyethyl methacrylate) scaffolds containing continuous pores prepared by the polymerization of 2-hydroxyethyl methacrylate (HEMA) and ethylene dimethacrylate (EDMA) in the presence of ammonium oxalate porogen. The scaffolds were thoroughly characterized by scanning electron microscopy (SEM), vibrating sample magnetometry, FTIR spectroscopy, and mechanical testing in terms of chemical composition, magnetization, and mechanical properties. While the SEM microscopy confirmed that the hydrogels contained communicating pores with a length of ≤2 mm and thickness of ≤400 μm, the SEM/EDX microanalysis documented the presence of γ-Fe_2_O_3_ nanoparticles in the polymer matrix. The saturation magnetization of the magnetic hydrogel reached 2.04 Am^2^/kg, which corresponded to 3.7 wt.% of maghemite in the scaffold; the shape of the hysteresis loop and coercivity parameters suggested the superparamagnetic nature of the hydrogel. The highest toughness and compressive modulus were observed with γ-Fe_2_O_3_-loaded PHEMA hydrogels. Finally, the cell seeding experiments with the human SAOS-2 cell line showed a rather mediocre cell colonization on the PHEMA-based hydrogel scaffolds; however, the incorporation of γ-Fe_2_O_3_ nanoparticles into the hydrogel improved the cell adhesion significantly. This could make this composite a promising material for bone tissue engineering.

## 1. Introduction

The application of magnetic biomaterials in combination with a magnetic field is an important issue in particular in regenerative medicine to control cell fate and investigate cell mechanotransduction [1,2]. Magnetically responsive polymer scaffolds are typically composed of magnetic nanoparticles incorporated within a three-dimensional polymer matrix based on both natural and synthetic polymers, such as collagen [3], alginate [4], gelatin [5], chitosan [6], poly(*N*-isopropylacrylamide) [7], poly(vinyl alcohol) [8], polydimethylsiloxane [9], or poly(ε-caprolactone-*b*-ethylene glycol-*b*-ε-caprolactone) [10]. Prominent among them are especially hydrophilic polymers exemplified by poly(2-hydroxyethyl methacrylate) (PHEMA). PHEMA-based materials have been widely used in contact and intraocular lenses, artificial cornea, vitreous humor replacement, artificial emboli, burn dressings, hemodialysis membranes, hemoperfusion packings, implants for soft tissue reconstructive surgery, reconstruction of vocal cord, etc. [11,12,13,14]. These applications were facilitated by suitable material properties, such as hydrophilicity (homogeneous PHEMA absorbs ~40% of water), swelling, biocompatibility, inertness, nondegradability, and tissue-like mechanical characteristics (softness) [15].

PHEMA hydrogels can be generally divided into several classes: nonporous, microporous (<2 nm pores), mesoporous (2–50 nm pores), macroporous (pore sizes ranging 50 nm–1 µm), and superporous (interconnected pores ~hundreds of µm in size) [16,17]. While the micro- to macropores are necessary for the transport of oxygen, nutrients, and/or removal of metabolites, superpores are important for passing large objects, mostly cells, e.g., blood cells, but also proteins, peptides, etc. Several methods have been described to design interconnected pore structures, including solvent casting and particulate leaching, freeze-drying, gas foaming, electrospinning, phase separation, etc. [18,19].

Parallelly with the design of new hydrogels, magnetic metal oxide nanoparticles can have a positive effect on cell adhesion and proliferation [20]. Hence, iron oxide nanoparticles are considered to be a good candidate for incorporation into a hydrogel due to their unique properties such as biocompatibility, small size, engineered surface, response to the external magnetic field, etc. In addition to magnetic nanoparticles, also nonmagnetic ones, exemplified by poly(L-lactide) or amino mesoporous silica, were combined with alginate-based hydrogels significantly improving the adhesion of the platelets or proliferation of human mesenchymal stem cells, respectively [21,22]. We have established an approach to introduce iron oxide nanoparticles in superporous PHEMA with large interconnected pores prepared with ammonium oxalate as a suitable porogen. This inert compound consists of needle-like crystals that allow parallel arrangement in the polymerization mold [23]. Ammonium oxalate needles thus act as a template that leaves pores after dissolving the salt during hydrogel washing. The pores in the magnetic nanoparticle-containing PHEMA scaffolds should allow for elastic reversible deformation using an external magnetic field. This ability could mechanically stimulate the cells cultivated on the scaffold, influencing tissue replacement during cell seeding. *In vitro* maturation and differentiation of the cells before the implantation might be thus affected, speeding up the healing process and improving the functionality of the scaffold soon after the implantation [24,25,26]. The possibility to deform the implant in a noninvasive way will also allow controlled perfusion of the cultivation medium under in vitro conditions. This is an important issue since the nutrition of cell components is a crucial problem often resulting in failure of the scaffold colonization by the cells [27]. Consequently, knowledge of the mechanical properties of hydrogels is significant in terms of maintaining their structural integrity during handing in tissue engineering applications.

To investigate the effect of magnetic polymer scaffolds on cellular behavior, three types of superporous PHEMA hydrogel scaffolds were prepared, including the magnetic one. The scaffolds were thoroughly physicochemically characterized and human osteoblastic cell line SAOS-2 was cultivated on them as an example of bone connective tissue to investigate cell adhesion and proliferation. The results suggested that the incorporation of γ-Fe_2_O_3_ nanoparticles in the hydrogel matrix considerably improved cell colonization. This makes the magnetic PHEMA-based scaffolds a promising candidate for applications in tissue engineering of connective tissues.

## 2. Materials and Methods

### 2.1. Materials

Ammonium oxalate, ferrous chloride tetrahydrate (FeCl_2_·4H_2_O), ferric chloride hexa-hydrate (FeCl_3_·6H_2_O), 2-hydroxyethyl methacrylate (HEMA), ethylene dimethacrylate (EDMA), 2,2′-azobis(2-methylpropionitrile) (AIBN), 2-dimethylaminoethyl methacrylate (DMAEMA), McCoy’s 5A medium, 4′,6-diamidino-2-phenylindol (DAPI), and Texas Red were purchased from Sigma-Aldrich (St. Louis, MI, USA). 1,4-Dioxan_,_ NH_4_OH, and NaCl were obtained from Lach-Ner (Neratovice, Czech Republic). Other reagents were supplied from Lachema (Brno, Czech Republic). Q-water ultrafiltered on a Milli-Q Gradient A10 system (Millipore, Molsheim, France) water was used in the experiments.

### 2.2. Preparation of γ-Fe_2_O_3_ Nanoparticles

Aqueous ferric chloride (0.2 M; 100 mL), ferrous chloride (0.2 M; 50 mL), and 0.5 M NH_4_OH (100 mL) were mixed using a Hielscher UP400St ultrasonic processor (Teltow, Germany) for 5 min. The solution was added to 0.5 M NH_4_OH (400 mL) and the mixture was stirred (250 rpm) for 1 h to yield magnetite nanoparticles. The particles were magnetically separated, washed with water five times, 5 wt.% sodium hypochlorite solution (16 mL) was added, and the mixture was sonicated for 5 min. The particles were again separated using a magnet and washed with water five times until peptization and γ-Fe_2_O_3_ formation [28].

### 2.3. Preparation of Nonmagnetic and Magnetic Superporous Poly(2-Hydroxyethyl Methacrylate) (PHEMA) and Poly(2-Hydroxyethyl Methacrylate-co-2-Dimethylaminoethyl Methacrylate) [P(HEMA-DMAEMA)] Hydrogels

**Figure 1:**
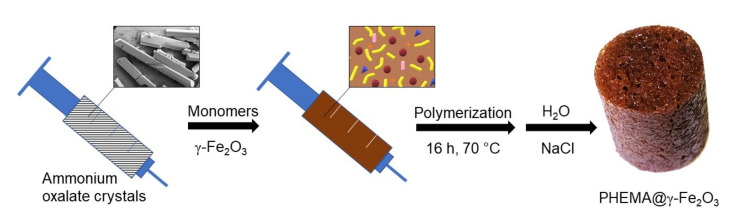
Scheme of preparation of a magnetic hydrogel scaffold.

The hydrogel scaffolds were prepared by a modification of an earlier published procedure [23]. Briefly, a solution of HEMA (3.1 g), optionally DMAEMA (120 mg and 2.98 g of HEMA), EDMA (33 mg), and AIBN (24 mg) in 1,4-dioxan (1.7 mL) was loaded in a 10-mL syringe filled with ammonium oxalate needle-like crystals (6.75 g; 41.8 vol.% of total volume) as a porogen and the mixture was polymerized at 60 °C for 16 h (Figure 1). After the reaction was completed, the hydrogel was removed from the syringe, the nonporous polymer above the crystals was cut off, and the scaffold was immersed in saturated NaCl solution for 24 h to avoid polymer cracking; the hydrogel was then washed in water for 10 days until all ammonium oxalate crystals were washed out. The magnetic hydrogel, denoted as PHEMA@γ-Fe_2_O_3_, was obtained similarly, but in the presence of γ-Fe_2_O_3_ nanoparticles (0.3 g) in the reaction feed.

### 2.4. Characterization of Hydrogels

Morphology of PHEMA, P(HEMA-DMAEMA), and PHEMA@γ-Fe_2_O_3_ hydrogels was visualized using a MAIA3 scanning electron microscope (SEM; TESCAN, Brno, Czech Republic) equipped with an X-Max^N^ 20 detector for energy-dispersive spectroscopy (EDX, Oxford Instruments, Abingdon, UK). The hydrogels were lyophilized, cut, and sputtered with a thin platinum layer before SEM observation. The micrographs were obtained by means of a secondary electron detector at an accelerating voltage of 3 kV. The EDX spectra (elemental microanalysis) were collected at an increased accelerating voltage of 30 kV.

Fourier transform infrared spectra (FTIR) were recorded using a Thermo Nicolet NEXUS 870 spectrometer (Madison, WI, USA) in the range of 600–4000 cm^–1^.

Magnetic properties of PHEMA@γ-Fe_2_O_3_ were determined with an EV9 VSM vibrating sample magnetometer (MicroSense, Lowell, MA, USA) at room temperature (RT) with the maximum applied magnetic field of 1 T. Hysteresis loops were measured with variable field steps, from 500 Oe (0.05 T) in the highest field range (300 mT–1 T) to the finest step of 5 Oe (0.5 mT) in the lowest field range (–25–25 mT) at the intersections with magnetization and field axes. This ensured determination of saturation remanent magnetization *M*_rs_ (i.e., magnetization at zero field—the intersection with the magnetization axis) and coercive force *B*_c_ (field necessary to induce zero magnetization—the intersection with the field axis). The two parameters were determined as average values of the corresponding intersections on the ascending and descending branches. Saturation-induced magnetization *M*_s_ was determined as the maximum induced magnetization after subtraction of the linear paramagnetic part of the loop. This was approximated by a linear fit of loop segment above 0.5 T, where the ferrimagnetic response to the external field is saturated and reflected by closed, reversible, and linear ascending and descending branches of the loop. Again, average *M*_s_ values were calculated. In order to check the ability of the magnetic hydrogel to acquire remanent magnetization, the magnetic field was step-wise increased, and remanence was measured after the field was switched off. After the maximum field (2 T) was applied, the hydrogel was assumed to retain saturation remanent magnetization. Finally, after the application of the maximum field, the same process was repeated, but with the field applied in the opposite direction. In this way, remanent magnetization was removed until zero value was reached. The opposite field, needed to remove *M*_rs_, is termed coercivity of remanence *B*_cr_ and, similar to *M*_rs_ and *B*_c_, also reflects maghemite particle size. Superparamagnetic particles behave similarly to paramagnetic atoms, i.e., they neither maintain stable remanent magnetization at RT nor show coercive force. Thus, both *M*_rs_ and *B*_c_ reflect particle size and are, in the case of superparamagnetic magnetite/maghemite, very close to zero [29]. Thus, magnetic measurements provide additional information on the concentration of maghemite/magnetite and prove its superparamagnetic character. Moreover, the possible role of interparticle interactions, which may result in collective behavior and clustering of maghemite/magnetite particles, may be assessed.

The internal pore structure of scaffolds was characterized on a Pascal 140 and 440 mercury porosimeter (Thermo Finigan, Rodano, Italy) operating in two pressure intervals (0–400 kPa and 1–400 MPa) and allowing determination of pore size in the range of 0.004–116 µm. The samples for measurement were prepared by cutting the hydrogel cylinders using a Leica DB80 LX microtome blade (Leica Biosystems, Nussloch, Germany) to small 5-mm cubes that were air-dried. The pore volume and most frequent pore diameter were calculated using the Pascal program and Washburn equation assuming a cylindrical pore model [30,31]. The volume of pores was evaluated as the difference between the end values on the volume vs. pressure curve. Porosity *p* (%) was calculated according to the formula: *p* = (*V* × 100)/(*V* + 1/ρ), where *V* was cumulative pore volume (cm^3^) and ρ was PHEMA density (ρ_PHEMA_ = 1.15 g/mL, ρ_PHEMA@γ-Fe2O3_ = 1.5 g/mL).

The static mechanical properties of the hydrogels were measured using an Instron 6025/5800R electromechanical testing machine (Instron, High Wycombe, UK) equipped with a 100 N load cell at a cross-head speed of 1 mm/min. The compression tests were performed on hydrogel cylinders (15.5 mm in diameter and 16 mm high) in water till reaching 50% deformation of the height. The values were averages of at least three measurements.

### 2.5. Tissue Culture Experiments

The PHEMA-based hydrogels were cut into 2 mm thick circular scaffolds that were placed into a 24-well tissue culture plate (TPP, Trasadingen, Switzerland) and sterilized from both sides for 10 min by UV-C irradiation from a germicidal lamp. SAOS-2 human osteoblastic cell culture (Cell Lines Service GmbH, Eppelheim, Germany) was cultivated in McCoy’s 5A medium with 15% fetal bovine serum (FBS) under standard cultivation conditions (37 °C and 5% CO_2_ atmosphere), seeded on the scaffolds at a density 10,000 cells/cm^2^, and subsequently cultivated for 1, 3, and 7 days.

### 2.6. Cell Metabolic Activity

The cell metabolic activity was measured using an MTS assay (Promega, Madison, WI, USA) based on yellow-colored 3-[4,5,dimethylthiazol-2-yl]-5-[3-carboxymethoxy-phenyl]-2-[4-sulfophenyl]-2H-tetrazolium inner salt (MTS) that changes to formazan (dark violet-red). The hydrogel scaffolds with adhered cells were transferred into a new 24-well cultivation plate, the MTS solution was diluted 1:5 v/v with cultivation medium in the absence of phenol red, and the cells were cultivated in the medium containing MTS for 2 h. Finally, the medium with metabolized tetrazolium salt (100 µL) was transferred to a 96-well plate in triplets and the amount of metabolized formazan was determined using a VersaMax microplate reader (Molecular Devices, San Jose, CA, USA) at 490 nm. Metabolic activity of SAOS-2 cells cultivated on polystyrene tissue culture plates served as a control.

### 2.7. Confocal Microscopy

First, the cells were fixed with 4% paraformaldehyde in phosphate-buffered saline (PBS) and the nuclei and actin cytoskeleton were stained by DAPI and Texas Red, respectively. The cells were then observed and photographed on a Dragonfly 503 confocal system with Zyla 4.2 PLUS sCMOS camera (Andor Oxford Instruments, Belfast, Ireland) mounted on a Leica DMi 8 microscope (Wetzlar, Germany) with 10× HC PL APO (N.A. = 0.40) objective. The confocal images were acquired at low magnification to view the overall coverage of cells on the hydrogel with a possibility to monitor cells deep inside of the scaffold. The maximum intensity projections were obtained using the Imaris software (Bitplane, Zürich, Switzerland).

## 3. Results and Discussion

### 3.1. Magnetic Superporous PHEMA Hydrogel Scaffolds

A simple approach to prepare a superporous polymer structure to mediate cell functions consisted of the copolymerization of HEMA with a small amount of EDMA crosslinking agent (1 wt.%), which prevents dissolution of the scaffold; the process took place in the presence of an insoluble crystalline material according to our well-established earlier procedure [23]. From a broad supply of various crystalline compounds, ammonium oxalate that forms needle-like crystals was chosen. Ammonium oxalate has additional advantages, such as easy purification and regulation of the crystallization process controlling, thus the size of crystals. This, in turn, defines the diameter of pores obtained after careful washing of the hydrogel with water. Optionally, HEMA and EDMA were polymerized with DMAEMA (3.9 wt.%) as a comonomer known for its pH- and also temperature-sensitive behavior that could be exploitable in theragnostic applications [32]. Moreover, γ-Fe_2_O_3_ nanoparticles prepared by a coprecipitation method were incorporated in some hydrogel matrices to render responsivity to a magnetic field. The schematic illustration of the synthesis of magnetic hydrogel is shown in Figure 1.

**Figure 2:**
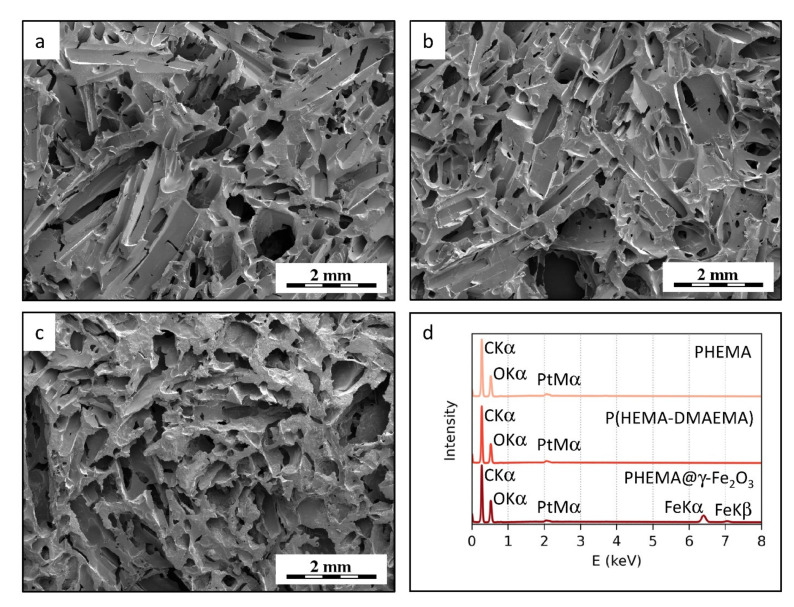
SEM micrographs of (**a**) PHEMA, (**b**) P(HEMA-DMAEMA), and (**c**) PHEMA@γ-Fe_2_O_3_ hydrogels, and (**d**) their EDX spectra that proved the presence of γ-Fe_2_O_3_ in the PHEMA@γ-Fe_2_O_3_ hydrogel.

Morphology and elemental composition of PHEMA, P(HEMA-DMAEMA), and PHEMA@γ-Fe_2_O_3_ hydrogels were assessed by SEM microscopy and microanalysis (Figure 2). All three materials exhibited almost identical microstructure. The hydrogel matrices contained communicating pores with length up to 2 mm and diameters up to 400 μm (Figure 2a–c) as a consequence of the fact that the hydrogels were prepared with the same ammonium oxalate porogen. Moreover, SEM/EDX spectra (elemental microanalysis) proved the chemical purity of the material due to the domination of carbon and oxygen peaks, with only a small amount of platinum (the samples were sputter-coated with Pt to increase contrast and minimize sample damage by the electron beam). SEM/EDX also confirmed the presence of γ-Fe_2_O_3_ in the PHEMA@γ-Fe_2_O_3_ hydrogel since clearly visible iron peaks were found in the spectrum (Figure 2d).

Both SEM micrographs and SEM/EDX spectra confirmed the homogeneous distribution of the γ-Fe_2_O_3_ in the PHEMA matrix. The SEM micrographs did not show any visible iron oxide aggregates, indicating that the nanoparticles were dispersed very homogeneously. Moreover, the SEM/EDX spectra showed that the same concentrations of iron were present in different scaffold locations.

**Figure 3:**
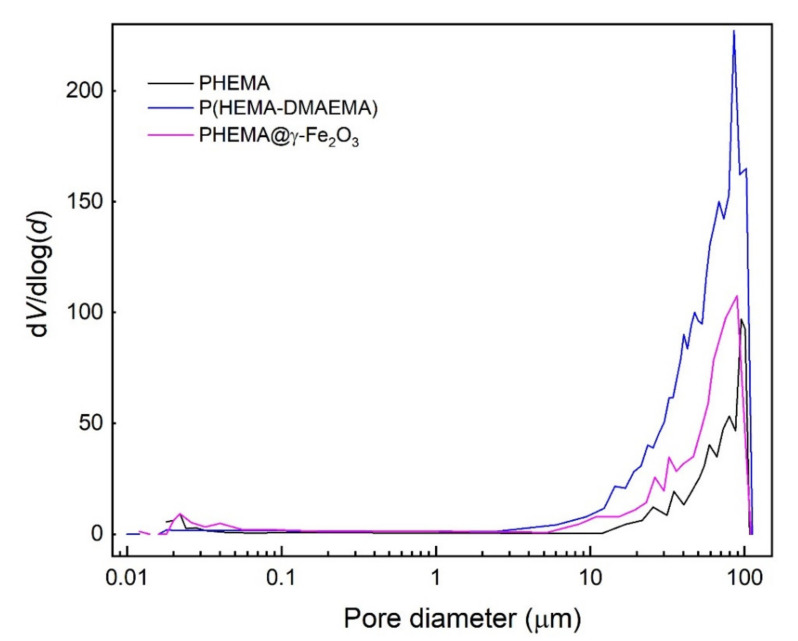
Pore size distribution of PHEMA, P(HEMA-DMAEMA), and PHEMA@γ-Fe_2_O_3_ hydrogels determined by mercury porosimetry; *V*–pore volume, *d*–pore diameter.

Macroporosity and partly mesoporosity of the PHEMA hydrogels were analyzed by a mercury porosimetry based on the penetration of mercury into the pores as a function of the applied pressure. The method covers the size distribution of pores ranging from 4 nm to 116 μm (Figure 3), as other pore sizes are not accessible to the mercury [30].

According to the measurements, the most frequent pore diameter of dry PHEMA and PHEMA@γ-Fe_2_O_3_ hydrogels varied between 84 and 93 μm (Table 1), which were larger values than those in poly(2-hydroxyethyl methacrylate-*co*-2-aminoethyl methacrylate) with the most frequent pore diameter of ~33 μm [23]. A larger pore size is convenient in terms of improved cell cultivation. Porosity or PHEMA and PHEMA@γ-Fe_2_O_3_ reached 22 and 32%, respectively, which were slightly lower values than observed earlier [23]. P(HEMA-DMAEMA) copolymer showed similar pore size (102 μm) but higher porosity (42%; Table 1). The reason for lower porosities of PHEMA and PHEMA@γ-Fe_2_O_3_ according to the mercury porosimetry, compared with the theoretical porosity corresponding to the amount of loaded ammonium oxalate (~42%), can be explained by the inability of the mercury porosimetry to detect the large superpores [30,33].

**Figure 4:**
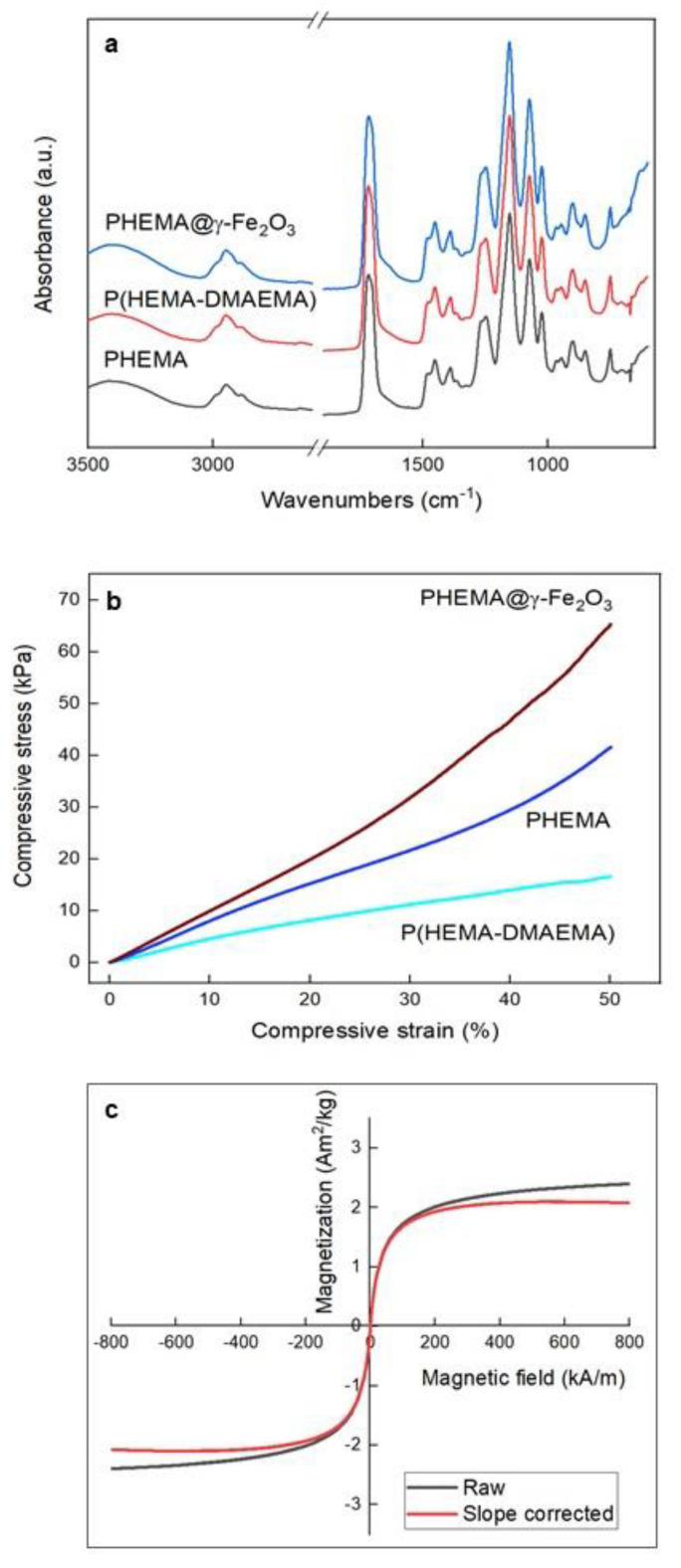
(**a**) FTIR spectra and (**b**) dependence of compressive stress of PHEMA, P(HEMA-DMAEMA), and PHEMA@γ-Fe_2_O_3_ hydrogels on strain, and (**c**) magnetic hysteresis loop of PHEMA@γ-Fe_2_O_3_ hydrogel both before and after correction for linear paramagnetic slope.

The chemical composition of PHEMA, P(HEMA-DMAEMA), and PHEMA@γ-Fe_2_O_3_ scaffolds was also investigated by FTIR spectroscopy (Figure 4a). In the spectra, the wide peak at ~3500 cm^–1^, with a maximum at 3415 cm^–1^, was recognized as the O–H stretching vibration. Three peaks at 2886, 2952, and 2989 cm^–1^ were assigned to the antisymmetric and symmetric stretching vibrations of CH_3_, CH_2_, and CH, respectively. The large peak at 1718 cm^–1^ was ascribed to the C=O stretching vibration, while the peak at 1454 cm^–1^ was attributed to the in-plane bending of CH_2_, the peak at 1390 cm^–1^ corresponded to the CH_2_ twist and rock vibration, and the peaks at 1074 and 748 cm^–1^ were attributed to the C–O–C stretching and −C–O– out-of-plane bending vibration, respectively. Peaks from P(HEMA-DMAEMA) seemed to be overlapped by stronger peaks of C–H and C=O bonds. The wide peak at ~600 cm^–1^ in the spectrum of PHEMA@γ-Fe_2_O_3_ was ascribed to Fe–O vibrational bands of iron oxide. The results of the FTIR analysis thus confirmed the presence of γ-Fe_2_O_3_ nanoparticles in the PHEMA structure.

Mechanical properties of the PHEMA, P(HEMA-DMAEMA), and PHEMA@γ-Fe_2_O_3_ hydrogels were determined in terms of compressive modulus, strength, and toughness (Figure 4b). Copolymerization of HEMA with DMAEMA, which enhanced both the pore size and porosity, had a significant effect on the decrease of mechanical properties up to 60%, compared to those of PHEMA (Table 1). The opposite trend was observed after the incorporation of the γ-Fe_2_O_3_ in the PHEMA matrix, where the mechanical properties increased up to 30%. Rather poor mechanical properties of P(HEMA-DMAEMA) hydrogel may be explained by its enhanced hydrophilicity associated with increased equilibrium swelling capacity [33].

Hysteresis loop of PHEMA@γ-Fe_2_O_3_ hydrogel, both before and after correction for linear paramagnetic slope, was shown with the field axis scaled from –0.5 to 0.5 T, although the loop was measured from –1 to 1 T (Figure 4c). The loop was very narrow, with low coercive force *B*_c_ (0.524 Oe; 5.24 × 10^–5^ T), reaching saturation at ~300 mT, which is the value of saturation field for magnetite or maghemite; the same applied to *M*_rs_ (3.61 × 10^–3^ Am^2^/kg). Saturation magnetization *M*_s_ was 2.037 Am^2^/kg, which in the case of multidomain maghemite would correspond to 2.55 wt.% of maghemite assuming its saturation magnetization ~80 Am^2^/kg [29]. However, magnetic parameters of synthetic nanosized magnetite are known to be size dependent, which can be used for comparison, assuming that both magnetite and maghemite are ferrimagnets of almost identical magnetic behavior [34]. Our observed values of *B*_c_ and *M*_rs_ of PHEMA@γ-Fe_2_O_3_ hydrogel were well below the values for 10 nm synthetic magnetite/maghemite. However, magnetite nanoparticles <20 nm should not be termed magnetite, because the oxidized shell surrounding the core prevails, and the fraction of maghemite increases [35]. Therefore, for our ~10 nm particles, *M*_s_ of ~55 Am^2^/kg can be used to estimate iron oxide concentration on the basis of *M*_s_ [34]. Considering that such small particles are rather a maghemite than magnetite [35], the estimated upper limit of maghemite concentration in PHEMA@γ-Fe_2_O_3_ (with *M*_s_ of 2.037 Am^2^/kg) was 3.7 wt.%, which is sufficient to be attracted by a magnet. Direct-field remagnetization revealed extremely soft behavior with a *B*_cr_ of 106.4 Oe (10.64 mT). All these data suggest that the particles were superparamagnetic. Moreover, such low values of *B*_c_, *B*_cr_, and *M*_rs_ suggest the absence of collective behavior (unwanted clustering) due to interparticle interactions. It should be also noted that PHEMA itself was diamagnetic, characterized by a weak and negative response to the applied magnetic field. On the other hand, maghemite was ferrimagnetic, with positive susceptibility (i.e., ability to respond to a magnetic field). This response was ~10,000 times higher than that for diamagnetic material. As a result, magnetic measurements sensed only maghemite, and the contribution of the hydrogel to magnetization would be visible as a slightly negative slope superimposed to the magnetization curve at very high fields, where maghemite was saturated.

### 3.2. In Vitro Testing of PHEMA-Based Hydrogels

**Figure 5:**
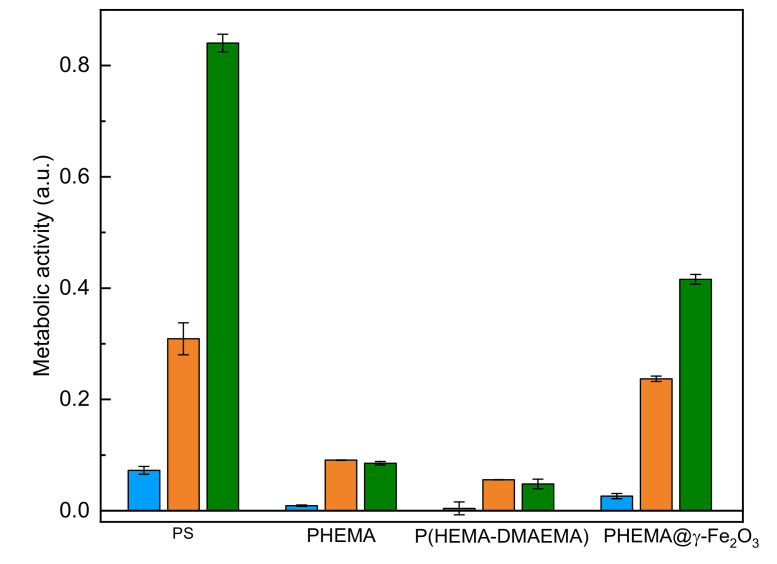
Metabolic activity of SAOS-2 cells cultivated for 1 (blue), 3 (orange), and 7 days (green) on poly(2-hydroxyethyl methacrylate) (PHEMA), poly(2-hydroxyethyl methacrylate-*co*-2-dimethylaminoethyl methacrylate) [P(HEMA-DMAEMA)], and PHEMA containing γ-Fe_2_O_3_ nanoparticles (PHEMA@γ-Fe_2_O_3_). PS—polystyrene culture plate.

In vitro biological experiments on PHEMA, P(HEMA-DMAEMA), and PHEMA@γ-Fe_2_O_3_ hydrogels were conducted with human osteoblast-like cells of SAOS-2 line, a representative of hard connective tissues. This cell type is used to evaluate material biocompatibility [36,37]. We used this cell type because one of the possible utilizations of the PHEMA-based materials is in bone tissue reconstruction. Cellular response to the hydrogels was investigated in terms of metabolic activity of SAOS-2 cells cultivated on the hydrogels for 1, 3, and 7 days (Figure 5). Enhanced metabolic activity, i.e., good cell proliferation, was observed in particular on PHEMA@γ-Fe_2_O_3_ hydrogel due to the favorable effect of magnetic particles on cell viability. With increasing time of cell cultivation, the metabolic activity on PHEMA@γ-Fe_2_O_3_ substantially increased (Figure 5). As expected, the metabolic activity of the cells grown on the PHEMA@γ-Fe_2_O_3_ scaffold did not reach the values on the plasma-treated polystyrene dish used as a control; moreover, the difference in the topography of flat tissue culture polystyrene plate and porous 3D PHEMA-based scaffold should be taken into consideration.

**Figure 6:**
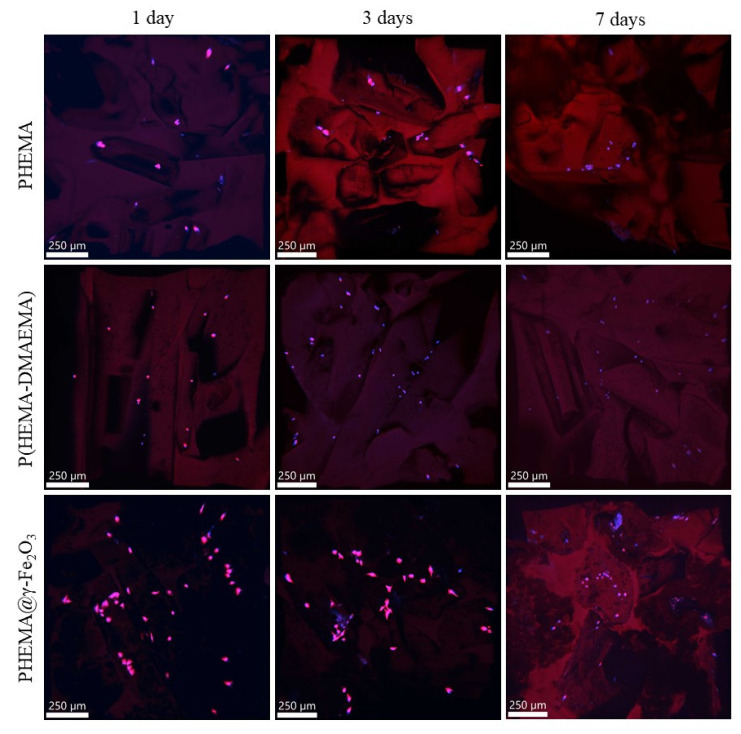
Confocal fluorescence micrographs of superporous PHEMA, P(HEMA-DMAEMA), and PHEMA@γ-Fe_2_O_3_ hydrogels after incubation with SAOS-2 cells for 1, 3, and 7 days.

While the adhesion of cells on the PHEMA and P(HEMA-DMAEMA) hydrogels was rather moderate with little spreading (data not shown), which agrees with other studies [38,39], the adhesion on PHEMA@γ-Fe_2_O_3_ was significantly improved. The enhanced cell adhesion to PHEMA@γ-Fe_2_O_3_ hydrogel, compared to other systems, can be explained by (i) the homogeneous dispersion of γ-Fe_2_O_3_ nanoparticles in the hydrogel matrix, (ii) affinity of the cells to iron, and/or (iii) changes in the surface topography (roughness) caused by the addition of γ-Fe_2_O_3_ nanoparticles. Moreover, the nanoparticles on the hydrogel increase the surface area, which, in turn, enhances the amount of adsorbed serum proteins. Further, changes in electrostatic interactions on the surface can affect the composition of the protein layer on the hydrogel and the conformation of adsorbed proteins [40]. These phenomena can explain higher cell numbers leading to increased cell metabolic activity on the scaffolds containing γ-Fe_2_O_3_; however, the improved cell metabolic activity can be also explained by the stimulating effect of iron oxide on cell energy metabolism [41]. Moreover, the porous structure of the hydrogels was visible in the confocal fluorescence micrographs due to a weak autofluorescence of the PHEMA matrix (Figure 6). The pore structure corresponded to the shape of original oxalate crystals that were loaded in the polymerization mixture during the synthesis of scaffolds. The cells were visible as bright dots or “polygons.” The confocal images showed that the SAOS-2 cells on PHEMA and P(HEMA-DMAEMA) hydrogels stayed in the thermodynamically least demanding round shape, whereas the cells on the PHEMA@γ-Fe_2_O_3_ spread into a typical polygonal shape demonstrating the supporting effect of γ-Fe_2_O_3_ on cell adhesion (Figure 6). This result is consistent with the finding that superparamagnetic iron oxide nanoparticles promoted endothelial progenitor cell adhesion [42]. The magneto-mechanical stimulation of the cells adhering to the PHEMA@γ-Fe_2_O_3_ scaffold is now in progress.

## 4. Conclusions

PHEMA belongs to the first synthetic hydrogels used in pharmaceutical applications. Porous PHEMA hydrogel scaffolds were then investigated in different biomedical applications, especially in orthopedics. Here, pore interconnectivity was essential for cell invasion and vascularization [43]. All the hydrogels prepared in this work, PHEMA, P(HEMA-DMAEMA), and PHEMA@γ-Fe_2_O_3_, exhibited similar morphology of mutually connected pores, as evidenced by SEM microscopy. The pores were anisotropic, with length up to 2 mm and thickness up to 400 μm. The matrix of the last hydrogel, PHEMA@γ-Fe_2_O_3_, contained γ-Fe_2_O_3_ nanoparticles, as confirmed by SEM/EDX microanalysis. The characterization of magnetic hydrogel scaffolds thus confirmed their successful formation. In mechanical measurements, high toughness and compressive modulus were achieved by the incorporation of γ-Fe_2_O_3_ into the PHEMA hydrogel matrix. In contrast, the toughness and compressive modulus of P(HEMA-DMAEMA) were rather mediocre probably due to the increased porosity and pores size of the material. The mechanical properties of the PHEMA-based materials are close to those of some types of living tissues. Although the cell adhesion on the PHEMA and P(HEMA-DMAEMA) hydrogels tested by the SAOS-2 cell line was modest, the cells were present on the scaffolds. Moreover, the cell adhesion was visibly improved by the incorporation of the γ-Fe_2_O_3_ nanoparticles into the PHEMA hydrogel matrix. This makes the magnetic superporous poly(2-hydroxyethyl methacrylate) hydrogel scaffold a promising precursor material, both for bone and/or other connective tissue engineering applications.

## Figures and Tables

**Table 1:** Porous and mechanical properties of PHEMA-based hydrogels measured by mercury porosimetry and compression test.

Hydrogel	Most Frequent Pore Diameter ^a^ (µm)	Pore Volume ^a^ (ml/g)	Porosity ^a^ (%)	Compressive Modulus (kPa)	Compressive Strength (kPa)	Toughness (mJ/mm^3^)
PHEMA	84	0.23	22	88.7 ± 15.9	46.5 ± 4.9	0.122 ± 0.02
P(HEMA-DMAEMA)	102	0.63	42	45.9 ± 8.5	16.5 ± 0.8	0.055 ± 0.01
PHEMA@γ-Fe_2_O_3_	93	0.40	32	98.5 ± 7.3	61.7 ± 8.0	0.154 ± 0.01

^a^ From mercury porosimetry; PHEMA–poly(2-hydroxyethyl methacrylate), P(HEMA-DMAEMA)–poly(2-hydroxyethyl methacrylate-*co*-2-dimethylaminoethyl methacrylate).

## Data Availability

Data is contained within the article.
